# Effects of different boiling processes on chemical compositions of *Lilii Bulbus* soup

**DOI:** 10.3389/fnut.2022.985105

**Published:** 2022-10-20

**Authors:** Guowei He, Ziying Liu, Hong Chen, Yuhui Wang, Wei Huang, Xiangyang Lu, Yun Tian, Huhu Liu

**Affiliations:** ^1^College of Bioscience and Biotechnology, Hunan Agricultural University, Changsha, China; ^2^State Key Laboratory of Utilization of Woody Oil Resource, Hunan Academy of Forestry, Changsha, China

**Keywords:** *Lilii Bulbus*, boiling process, total polysaccharide, heavy metal, edible Chinese herb medicine

## Abstract

*Lilii Bulbus*, an edible Chinese herbal medicine, has a long history in medicine. However, research on effectively boiling *Lilii Bulbus* is rare. To make the more nutritious *Lilii Bulbus* soup, the optimized boiling process, using an alternate heating mode by decoction pot carrying a mixture of water and Chinese liquor at the ration of 9:1, was established in this study. Compared to the soup prepared by the daily process, the polysaccharide amount improved by 54%, and the total heavy metals decreased by 33.5% using the optimized boiling process. In addition, the total saponins at 34.3 μg/g were determined in the soup prepared by the optimized process. Meanwhile, the colchicine content in the boiled *Lilii Bulbus* soup was undetectable using the optimized process. This research performs an optimized boiling process for making *Lilii Bulbus* soup, and provides a reference for generating high commercial value from *Lilii Bulbus* soup in the future.

## Introduction

*Lilii Bulbus*, an edible Chinese herbal medicine recognized by the China Food and Drug Administration (CFDA), is the fleshy bulb scale of *Lilium brownii F.E. Brown var. viridulum Baker* from the family *Liliaceae*. *Lilii Bulbus* contains many active components, including polysaccharides, steroidal saponins, and alkaloids, which perform clinical functions, such as relieving cough and asthma, reducing blood sugar, and improving immunity (1). Among these, polysaccharides are one of the major components of *Lilii Bulbus* because they exhibit anti-tumor, blood lipid reduction, anti-viral, and anti-mutation functionalities, and also improve cellular immune activity (2, 3). *Lilii Bulbus* is also particularly rich in steroidal saponins which exhibit anti-inflammatory, antioxidant, antidepressant, and anti-tumor effects (4, 5).

*Lilii Bulbus* is cultivated in China and has a long history of being consumed locally as food (6). In particular, *Lilii Bulbus* cultivated in Hunan, Jiangsu, Zhejiang, and Gansu is of high quality and is beneficial to humans. At present, boiling is the most common method used to cook *Lilii Bulbus* in China, such as in its frequent use in Cantonese slow-cooked soups (7). However, the structure of the nutritional components is subject to change by the cooking method, resulting in their loss and destruction (8, 9).

Presently, the food chain is readily contaminated because of the bioaccumulation, lack of degradability, and excessive presence of heavy metals (10). Long-term intake and accumulation of such heavy metals in the blood can be very harmful to human health (11–13). Previously, the National Poisons Unit in London conducted a pilot survey to investigate the frequency of occurrence and the severity of adverse effects and toxicity from exposure to traditional medicines and food supplements. In this survey, five confirmed cases of heavy metal poisoning resulting from the use of contaminated traditional herbal remedies were found (14). Exogenous contaminants, such as the previously discussed heavy metals, are inevitably found in *Lilii Bulbus* because it grows underground (15). In previous studies, cadmium (Cd) content exceeded the safe values in *Lilii Bulbus* samples from Zhejiang (16). More recently, the contents of Cd, copper (Cu), and mercury in 60 commercial Chinese medicinal materials, including *Lilii Bulbus*, were reported to exceed the limits of the “Green Trade Standards of Importing and Exporting Medicinal Plants and Preparations” (WM2-2001). Cd, mercury, and Cu content in these herbal medicines exceeded safe limits by 38.8, 8.3, and 1.7%, respectively (17). Thus, it is obvious that in relation to functional nutritional content and heavy metal contamination, the boiling methods used in the preparation of soup need further investigation.

Numerous studies have been conducted on the characteristics of the nutrients in soups in relation to different boiling processes. For example, Song et al. (18) investigated the effects of different boiling times on *Bian-Que Triple-Bean Soup*. In this study, the total content of phenolics, saponins, tannins, and monomeric anthocyanins in the soup were determined, and the best boiling time that also retained the active ingredients was found. Additional auxiliary materials are also commonly added during cooking and can have a desirable effect on the active elements discussed previously. Liquor or vinegar is often used to process medicinal materials in the pharmacopoeia (1). For example, liquor has been used to reduce the bitter taste of *Angelica dahurica* (19). Silva et al. (20) found that vinegar can be used to improve the preservation capability of pickled jurubeba fruits. However, the researchers have focused on the desirable qualities of *Lilii Bulbus* (21, 22); research on effectively boiling *Lilii Bulbus* is rare.

To prepare more nutritious *Lilii Bulbus* soup based on the retention of active components (polysaccharides and saponins) and the reduction of harmful substances (heavy metals and colchicine), this study aimed to establish a more suitable boiling process by optimizing the heating mode and boiling solvent used. These conditions were varied in a series of boiling processes which were compared to the daily process. Thus, this research will provide an optimized boiling process for making *Lilii Bulbus* soup in daily life and also offers the reference for generating a higher commercial value for this soup.

## Materials and methods

### Preparation of materials

*Lilii Bulbus* was purchased from Beijing QianCao Chinese Herbal Pieces Co., Ltd. Chinese liquor (38% concentration) was purchased from Beijing Shunxin Agriculture Co., Ltd. White vinegar was purchased from Changsha Xianglian Sauce and Food Co., Ltd. The total polysaccharide were measured by the Total Polysaccharide Determination Kit (KeMing, China). The decoction pot was purchased from Joyoung Co., Ltd. The digital electric heater was purchased from Shanghai LiChen Instrument Technology Co., Ltd.

### The different boiling processes of *Lilii Bulbus*

Fifty gram *Lilii Bulbus* were weighed out and soaked in 500 mL solvent with a 1:10 material to liquid ratio for 30 min.

Daily process: Pure water was used as the solvent, and a decoction pot was used to boil the *Lilii Bulbus*. The high heat mode was applied to boil for 30 min, and then the gentle fire mode was applied to boil for 20 min. The obtained soup was filtered to obtain the supernatant, 500 mL of solvent was added again, and *Lilii Bulbus* was boiled in the same manner to obtain the second soup. The final soup was a mixture of the two supernatants.

Process 1: Pure water was used as solvent, and a digital electric heater was used to boil *Lilii Bulbus*. The 100°C mode was applied to boil the soup for 30 min. The boiled soup was filtered to obtain the supernatant, 500 mL of solvent was added again, and *Lilii Bulbus* was boiled in the same process to obtain the second soup. The final soup was a mixture of the two supernatants.

Process 2: Using a mixture of water and Chinese liquor in a 9:1 ratio as the solvent, the digital electric heater was used to boil *Lilii Bulbus*. The 100°C mode was applied to boil for 30 min. The boiled soup was filtered to obtain the supernatant, 500 mL of solvent was added again, and *Lilii Bulbus* was boiled in the same manner to obtain the second soup. The final soup was a mixture of the two supernatants.

Process 3: Using a mixture of water and Chinese liquor in a 9:1 ratio as the solvent, a decoction pot was used for boiling *Lilii Bulbus*. The high heat mode was applied to boil the soup for 30 min, and was then switched to the gentle fire mode to boil for 20 min. The boiled soup was filtered to obtain the supernatant, 500 mL of solvent was added again, and *Lilii Bulbus* was boiled in the same manner to obtain the second soup. The final soup was a mixture of the two supernatants.

Process 4: A mixture of water and white vinegar in a 9:1 ratio was used as the solvent, and a digital electric heater was used for boiling *Lilii Bulbus*. The 100°C mode was applied to boil the soup for 30 min. The boiled soup was then filtered to obtain the supernatant, 500 mL of solvent was added again, and *Lilii Bulbus* was boiled in the same process to obtain the second soup. The final soup was a mixture of the two supernatants.

Process 5: A mixture of water and white vinegar in a 9:1 ratio was used as the solvent, and a decoction pot was used to boil the *Lilii Bulbus*. The high heat mode was applied to boil the soup for 30 min, after which the gentle fire mode was used to boil it for another 20 min. The boiled soup was filtered to obtain the supernatant, 500 mL of solvent was added again, and *Lilii Bulbus* was boiled in the same manner to obtain the second soup. The final soup was a mixture of the two supernatants.

The boiled soup was kept at a constant volume of 500 mL and filtered using a 0.22 μm membrane for analysis in all processes.

### Extraction and determination of polysaccharide content

The total polysaccharide content of *Lilii Bulbus* was extracted in accordance with a previously reported method (23), which involved extracting the polysaccharide with water and precipitating it using alcohol. The amount obtained was tabulated using the phenol-sulfuric acid method. 1 mL of soup was used for this procedure to which 1 mL of water was added to dissolve the polysaccharide, 200 μL of which was absorbed. 100 μL of Reagent 1 and 0.5 mL concentrated sulfuric acid were added to the product, which was kept in a water bath at 90°C for 20 min. Running water was used to cool the product after this step. The absorbance value A was determined at a wavelength of 490 nm. Polysaccharide content was determined according to the manufacturer’s instructions.

### Analysis of heavy metal content

A boiling tube containing 1 mL of the soup and 10 mL of acid mixture (GR grade HNO_3_: GR grade HClO_4_ = 4:1) was held at 25°C for approximately 16 h. It was then heated to 95°C and held at that temperature for 2 h using a water bath, and was removed when the digested sample was transparent and had a volume of approximately 1 mL. The cooled volume was fixed at 25 mL using pure water before filtering. The digested samples were analyzed using Inductively Coupled Plasma Mass Spectrometry (ICP-MS).

### Extraction and determination of saponins content

The total saponin content of *Lilii Bulbus* and its presence in the soup was determined using vanillin-perchloric acid colorimetry, which has been reported previously (24). A 5 mg/mL vanillin-glacial acetic acid test solution and a 0.2 mg/mL oleanolic acid standard solution were prepared. The solvent of the soup was completely volatilized at 80°C, after which 50 μL vanillin-glacial acetic acid and 200 μL perchloric acid were added to it. It was then placed in a water bath at 70°C for 15 min, and then cooled with a cold water bath for another 5 min. Thereafter, 650 μL glacial acetic acid was added and the mixture was shaken well and allowed to stand for 10 min. The absorbance value A was determined at a wavelength of 540 nm.

### Detecting colchicine in the prepared soup

The presence of colchicine in soup was determined by High-Performance Liquid Chromatography (HPLC) under the following working conditions: Column: GL C18 (150 mm × 4.6 mm, 5 μm). Mobile phase: methanol: water (40:60, V: V) and isocratic elution. Flow rate: 1.0 mL/min. Determination wavelength: 254 nm. Column temperature: 30°C. Injection volume: 10 μL.

### Statistical analyses

The raw data were processed using Microsoft Excel (Microsoft, USA). Statistical analyses and charting were performed using OriginPro 2021 (OriginLab, USA). Pairwise comparisons were analyzed using Student’s *t*-test. Differences were considered statistically significant at *p* < 0.05. All reported results were the average of three identical experimental runs.

## Results and discussion

### Effects of different boiling processes on total polysaccharides content in *Lilii Bulbus* soup

**FIGURE 1:**
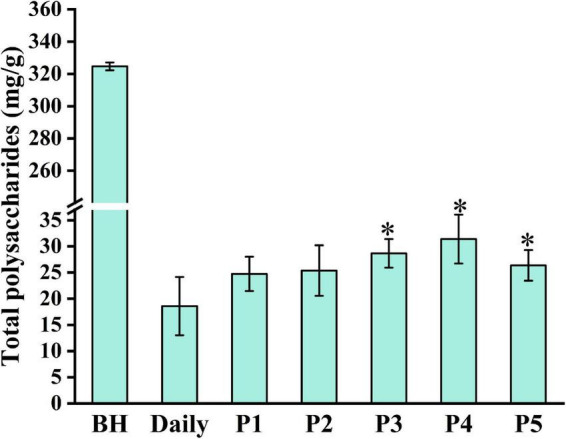
Total polysaccharide content in *Lilii Bulbus* and its soup. BH, *Lilii Bulbus*; Daily, Daily process; P1, Process 1; P2, Process 2; P3, Process 3; P4, Process 4; P5, Process 5. Significant differences between Daily and other processes (Student’s *t*-test): *P < 0.05.

Polysaccharides are important active components of traditional Chinese medicine. To evaluate the boiling effects of different boiling processes, the total polysaccharide content in *Lilii Bulbus* and its soup under different boiling processes was analyzed. As shown in Figure 1 and Table 1, an 18.6 mg/g concentration of polysaccharide was present in the boiled soup prepared by the daily process, accounting for 5.7% of total polysaccharide content in *Lilii Bulbus*. This indicates that there is still considerable potential to further optimize the boiling of *Lilii Bulbus* soup. Therefore, we optimized the solvent and heating methods to obtain a higher polysaccharide content in the soup of *Lilii Bulbus*. The best polysaccharide extraction was achieved by process 4, in which the polysaccharide content of the soup was 31.4 mg/g (*p* < 0.05), which is approximately twice that of the soup prepared by daily processing. It should be noted that white vinegar was added as an additive solvent and an electric heater was used to maintain boiling in this process. In addition, the polysaccharide content of the soup prepared by process 3 reached 28.7 mg/g (*p* < 0.05). Furthermore, we found that the extraction of polysaccharides in soups prepared by the boiling processes that used an additive (processes 2, 3, 4, and 5) was generally better than when using only pure water (daily process and process 1), regardless of the type of additive used.

**TABLE 1:** Compositions and content of *Lilii Bulbus.*

Material	Compositions	Content (μ g/kg)
*Lilii Bulbus*	Polysaccharide	3.246 × 10^8^
	As	202.4
	Pb	1460.5
	Cd	878.0
	Cr	8478.7
	Cu	56.0
	Saponin	6.322 × 10^5^
	Colchicine	29.4

Modern pharmacological studies show that polysaccharides from the traditional Chinese medicine have anti-tumor, immunity enhancing, anti-inflammatory, and anti-oxidation effects as well as helping to regulate the intestinal microenvironment (25–29). It is shown that polysaccharide can easily be depolymerized during the extraction process, and starch can easily be gelatinized and denaturalized (30). For example, garlic, like *Lili Bulbus*, is a plant of *Liliaceae*, which is also rich in polysaccharides. Lu et al. (31) found that high temperatures promote the degradation of high-MW polysaccharides into low-MW oligosaccharides and monosaccharides during black garlic processing. This phenomenon may explain why the boiled soup contains fewer polysaccharides than *Lilii Bulbus* itself. Moreover, various polysaccharides coexist in natural materials (32). Different extracting solvents, such as aqueous solutions with different concentrations of acid or alkali and temperatures, are used to separate polysaccharides with special properties (33–36). In addition to the water-soluble polysaccharides that are often studied, the alcohol-soluble polysaccharides in the traditional Chinese medicine are rarely studied (37, 38). The Chinese liquor was added to the solvent of process 2 and process 3, and some alcohol-soluble polysaccharides may be extracted. This may be why the content of polysaccharides in the soup prepared by process 2 and process 3 is higher than that of the daily process.

### Effects of different boiling processes on heavy metal content in *Lilii Bulbus* soup

Heavy metals with significant biological toxicity, such as Cd, lead (Pb), chromium (Cr), and arsenic (As), can harm humans (39, 40). Herein, five common heavy metals in *Lilii Bulbus* itself and its soup, including As, Pb, Cd, Cr, and Cu, were analyzed.

#### Arsenic

**FIGURE 2:**
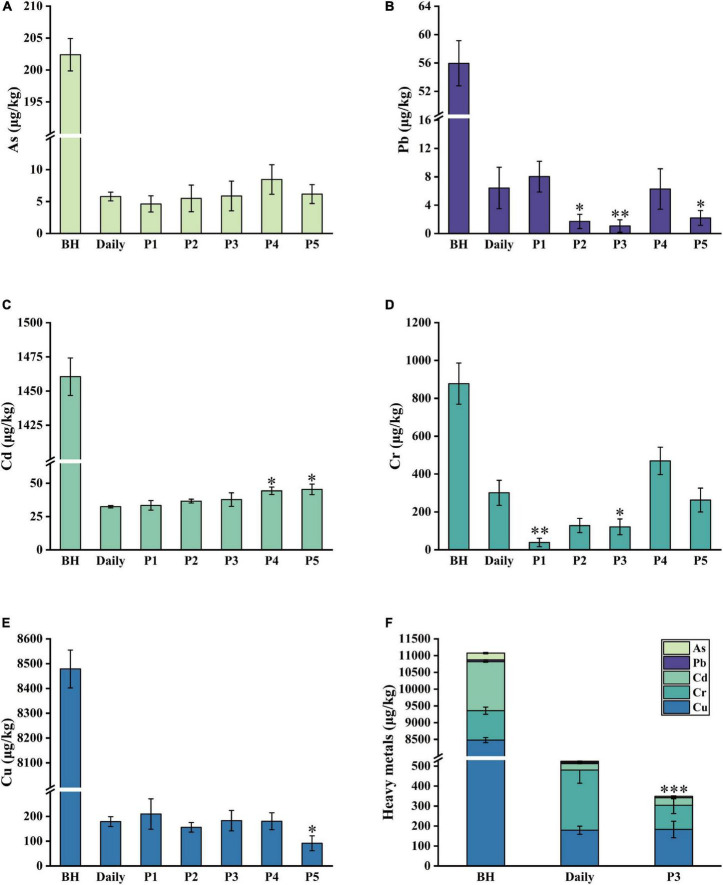
Five heavy metals content in *Lilii Bulbus* and its soup. **(A)** As. **(B)** Pb. **(C)** Cd. **(D)** Cr. **(E)** Cu. **(F)** Total heavy metals content in the soup prepared by optimized process. BH, *Lilii Bulbus*; Daily, Daily process; P1, Process 1; P2, Process 2; P3, Process 3; P4, Process 4; P5, Process 5. Significant differences between Daily and other processes (Student’s *t*-test): *P < 0.05, ^**^P < 0.01, ^***^P < 0.001.

As is the major pollutant in soil and water, and easily finds its way into the food chain through plants (41). The change of As content in the soup corresponding to the different boiling treatments is shown in Figure 2A. Compared to *Lilii Bulbus*, the *Lilii Bulbus* soup prepared by process 1 exhibited the best removal of As. The As content in the soup prepared by process 1 was 4.6 μg/kg, which was 19.3% lower than that in the soup prepared by daily process (5.7 μg/kg). Process 2 and process 3 exhibited 5.5 and 5.9 μg/kg of As, respectively. The results showed no significant improvement on the daily process. In addition, the addition of white vinegar showed no advantage regarding the removal of As.

#### Lead

As shown in Figure 2B, Pb removal from the soup was also significantly affected by the different boiling processes. In particular, boiling process 3 performed the best, and reduced the Pb content in the soup significantly to 1.1 μg/kg, which was 98.1% lower than that of the daily process (56.0 μg/kg, *p* < 0.01). The Pb content in the soup prepared by process 2 was 1.7 μg/kg, which is 97% lower than that of the daily process (*p* < 0.05). Thus, process 2 was second only to process 3. In addition, process 5 had a desirable impact on the removal of Pb from boiled soup. As shown in Figure 2B, under boiling process with different solvents, the Pb content of the boiled soup prepared by continuous boiling at 100°C was higher than that of the soup prepared by boiling via high heat and gentle fire.

#### Cadmium

As shown in Figure 2C, the Cd content in *Lilii Bulbus* was 1460.5 μg/kg and in the soup prepared by the daily process it was 32.5 μg/kg. The Cd removal effects of process 1 (33.5 μg/kg), 2 (36.7 μg/kg), and 3 (37.8 μg/kg) were similar to that of the daily process, with no significant difference between them. This demonstrates that the Cd content was not removed more efficiently by the different boiling processes. The Cd content in the soup prepared by 100°C continuous boiling was similar to that of the soup prepared by high heat mode and gentle fire mode, regardless of the type of solvent used (Figure 2C). Thus, optimization of the heating method and boiling time may have little effect on Cd removal. Nevertheless, compared with that in *Lilii Bulbus* (878.0 μg/kg, Table 1), the Cd content in the boiled soup was decreased by more than 96.8%.

#### Chromium

There was a clear change observed in the Cr content compared to the soup prepared by the daily process. As shown in Figure 2D, process 1 showed the best removal of Cr. The Cr content in the resulting soup was found to be 38.7 μg/kg, which was 87.1% lower than that of the daily process (301.1 μg/kg, *p* < 0.01). Process 3 was the second most effective and the Cr content in the soup made by process 3 was found to be 121.2 μg/kg, 59.7% lower than that in the soup prepared by daily processing (*p* < 0.05). These results are consistent with the trend observed for As removal.

#### Copper

As shown in Figure 2E, the minimum Cu content, 91.5 μg/kg, was demonstrated in the soup prepared by process 5. This was 48.9% lower than that in the soup prepared by daily process (179.0 μg/kg, *p* < 0.05), and the optimized effect was remarkable. The subsequent most effective process was process 2 (156.2 μg/kg). However, it achieved a Cu content only 12.8% lower than that in the daily process. The effects of processes 3 and 4 were similar to those of the daily process, and the difference was not significant.

Based on the above content analysis, five heavy metals were found to accumulate in *Lilii Bulbus* at different levels (Table 1). Among them, Cu was the most abundant in *Lilii Bulbus*, which may be because Cu is an essential nutrient for plants (42). Researchers have also used boiling to bring about a significant reduction in pesticide residues in pepper fruit (43). Here, our results showed that boiling is also effective for the removal of heavy metals. Korfali et al. (44) investigated the changes of heavy metal content in a variety of local herbs. In this study, it was found that the contents of Cr, Pb, and As in the boiled decoction are lower than those in the soaked decoction, while it was only boiled with pure water for 5 min. Arpadjan et al. (45) also showed that the contents of As, Cd, and Pb in the herbal decoction are significantly lower than those in the herbs themselves.

The As, Pb, Cd, Cr, and Cu contents in the soup prepared by processes 2 and 3 were significantly lower than those of *Lilii Bulbus* and the soup prepared by the daily process. In particular, process 3 reduced the total heavy metal content in the boiled soup by 33.5% (Figure 2F). This suggests that the addition of Chinese liquor for boiling is beneficial for heavy metal removal, and a similar trend may be observed in general herbal medicine. Ajaiyeoba et al. (46) found that boiling in water or alcohol is the most commonly used method to prepare drug decoctions. Yi et al. (47) also found that the main components, including chlorogenic acid and syringoside, in the alcohol extract of *Saussurea laniceps* are higher than those in the water extract, and the alcohol extract was also more effective in pharmacological tests. In addition, the study showed that processing with rice wine can significantly reduce the cytotoxicities and enhance the anti-inflammatory effects of *Herba Siegesbeckiae* (48).

**FIGURE 3:**
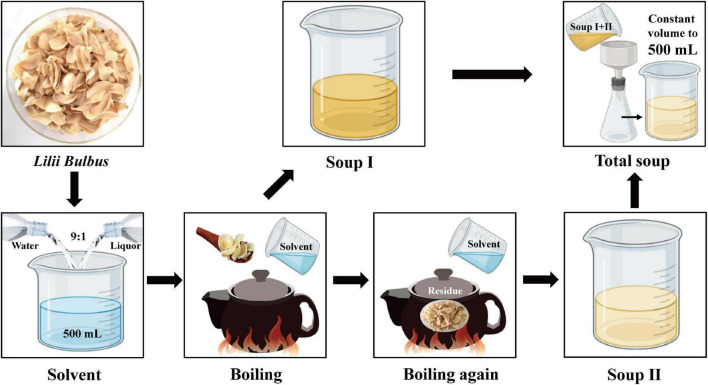
The operation flowchart of process 3.

According to the first edition of general rule 0212 of the Chinese Pharmacopoeia (2020 Edition), the contents of Pb, Cd, As, Hg, and Cu in medicinal materials and decoction pieces (plants) should not exceed 5, 1, 2, 0.2, and 20 mg/kg, respectively (1). The Joint FAO/WHO Expert Committee for Food Additives (JECFA) reported that the weekly allowable intake of Pb, As, Cd, and Hg was 25 μg/kg body weight (BW) (49), 15 μg/kg BW (50), 7 μg/kg BW (51), and 5 μg/kg BW (49), respectively, and the maximum allowable intake for healthy adults weighing 60 kg was 14 μg/d for Pb, 129 μg/d for As, 60 μg/d for Cd, and 43 μg/d for Hg (52). The heavy metal content in *Lilii Bulbus* soup prepared using processes 2 and 3 was demonstrated to be far below these limits, respectively. Moreover, the polysaccharide content in the soup prepared using process 3 was higher than that in the soup prepared using process 2. Process 3 was thus found to be the most optimized process with the most desirable effects in our study. The procedure of process 3 is shown in Figure 3.

### Effects of the optimized process on total saponins content in *Lilii Bulbus* soup

**FIGURE 4:**
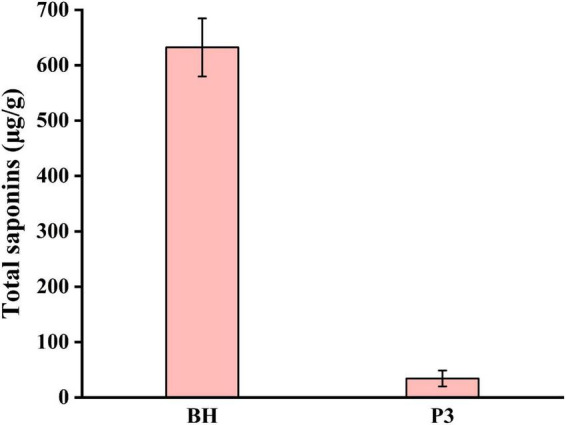
Total saponins content in the *Lilii Bulbus* soup prepared by process 3. BH: *Lilii Bulbus*, P3, Process 3.

Saponins in *Lilii Bulbus* are steroidal saponins, including spirostanol saponins (53), isospirostanol saponins (54), deformed spirostanol saponins (55), and furostanol saponins (56), which perform a variety of pharmacological functions (57, 58). To evaluate the effect of process 3 on the total saponins, the saponin content in *Lilii Bulbus* soup prepared by process 3 was determined. As shown in Figure 4, the total saponin content in the soup obtained was only 34.3 μg/g, whereas the total saponin content in *Lilii Bulbus* itself was 632.2 μg/g (Table 1). Thus, nearly 94.6% of the saponins could not be retained in the soup after boiling, indicating that process 3 could be further optimized for saponin retention, even though it was the most effective in terms of the extraction of polysaccharides and the removal of heavy metals.

There are two reasons that may lead to a low extraction rate of saponins in the soup. Firstly, saponins are steroid or triterpenoid glycosidic compounds that are not highly water-soluble, therefore, the saponins leached from the tissue would be minimal. Secondly, saponins are often lost when the raw materials are processed. For example, significant decreases in the total saponin content and the content of the three major saponin components were observed following soaking and canning of dry beans (59). Similar losses have been noted in the saponin content of moth beans that have undergone pressure-cooking (60). This phenomenon is similar to our results. Choi et al. (61) studied the contents of crude saponin in decoctions of *Platycodi Radix* prepared using different extraction processes. In this study, it was found that the crude saponin content in the decoctions increase with increasing extraction time, volume of water, extraction temperature, and number of repetitions for extraction.

### Effects of the optimized process on colchicine content in *Lilii Bulbus* soup

**FIGURE 5:**
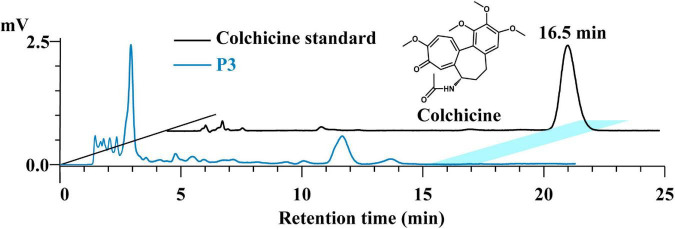
HPLC analysis of the colchicine in the *Lilii Bulbus* soup prepared by process 3. P3, Process 3.

Colchicine is a botanical alkaloid originally extracted from the seeds and bulbs of *Colchicum autumnale L* and is also found in *Lilii Bulbus*. The colchicine content in the soup prepared by process 3 was determined to evaluate its specific leaching effect. As shown in Figure 5, colchicine was not detected in the soup. In contrast, the colchicine content of *Lilii Bulbus* was 29.4 μg/kg (Table 1). This demonstrates that colchicine in *Lilii Bulbus* will not be extracted after boiling according to process 3, and there are no concerns about colchicine dosing when consuming the *Lilii Bulbus* soup.

Colchicine has been used to treat and prevent gout attacks and, in the past decade, the properties of colchicine was explored for use in the secondary prevention of cardiovascular diseases (62). Colchicine is most commonly administered orally, formulated in tablets of 0.5 or 0.6 mg. Intravenous administration is also possible in the form of a 0.5 mg/mL solution. However, gastrointestinal side effects occur in up to 80% of the patients receiving full therapeutic doses of colchicine (63). Therefore, to take in as little colchicine as possible when the dosage is uncertain, its retention should be minimized in the *Lilii Bulbus* soup. Here, our result was consistent with our view. In fact, colchicine may combine with the acidic components of plants and be found as salts in them because of its polarity and alkalinity, and the *Lilii Bulbus* must accordingly be alkalified before extraction to allow colchicine to exist as a free alkali (64, 65). Our boiling process does not include the alkalization step, and this may be the reason why we failed to detect colchicine in the boiled soup.

## Conclusion

In this study, we optimized the boiling process for making *Lilii Bulbus* soup, focusing on the heating method and the boiling solvent. The best effects on boiling polysaccharide of *Lilii Bulbus* and removing five heavy metals were shown under process 3, resulting in 28.7 mg/L of polysaccharide and 0.349 mg/kg of five heavy metals in total in the boiled soup. Compared to the soup prepared by the daily process, the polysaccharide amount improved by 54%, and the total heavy metals decreased by 33.5%. In addition, 34.3 μg/g of total saponins was determined in the soup prepared by the optimized boiling process 3. Meanwhile, the colchicine content in boiled *Lilii Bulbus* soup was undetectable using the optimized process. This study explored the characteristics of *Lilii Bulbus* soup under different boiling processes, focusing on the retention of polysaccharides and removal of heavy metals. More factors, including color and phytochemical composition, can be considered to comprehensively improve the quality of soup. Together, this research provides a reference for making commercial *Lilii Bulbus* soup via optimizing the boiling process of *Lilii Bulbus*.

## Data availability statement

The original contributions presented in this study are included in the article/supplementary material, further inquiries can be directed to the corresponding author/s.

## Author contributions

GH: conceptualization, methodology, data curation, and writing-original draft. ZL: conceptualization, methodology, and supervision. HC: validation and formal analysis. YW: formal analysis. WH: investigation. XL: data curation. YT: writing-reviewing and editing and funding acquisition. HL: funding acquisition, writing-reviewing and editing, and project administration. All authors contributed to the article and approved the submitted version.
